# Unraveling interindividual variation of trimethylamine *N*‐oxide and its precursors at the population level

**DOI:** 10.1002/imt2.183

**Published:** 2024-03-30

**Authors:** Sergio Andreu‐Sánchez, Shahzad Ahmad, Alexander Kurilshikov, Marian Beekman, Mohsen Ghanbari, Martijn van Faassen, Inge C. L. van den Munckhof, Marinka Steur, Amy Harms, Thomas Hankemeier, M. Arfan Ikram, Maryam Kavousi, Trudy Voortman, Robert Kraaij, Mihai G. Netea, Joost H. W. Rutten, Niels P. Riksen, Alexandra Zhernakova, Folkert Kuipers, P. Eline Slagboom, Cornelia M. van Duijn, Jingyuan Fu, Dina Vojinovic

**Affiliations:** ^1^ Department of Genetics, University Medical Center Groningen University of Groningen Groningen The Netherlands; ^2^ Department of Pediatrics, University Medical Center Groningen University of Groningen Groningen The Netherlands; ^3^ Department of Epidemiology Erasmus University Medical Center Rotterdam The Netherlands; ^4^ Metabolomics & Analytics Centre, Leiden Academic Center for Drug Research Leiden University Leiden The Netherlands; ^5^ Molecular Epidemiology, Department of Biomedical Data Sciences Leiden University Medical Center Leiden The Netherlands; ^6^ Department of Laboratory Medicine, University Medical Center Groningen University of Groningen Groningen The Netherland; ^7^ Department of Internal Medicine and Radboud Institute for Molecular Life Sciences Radboud University Medical Center Nijmegen The Netherlands; ^8^ Department of Internal Medicine Erasmus University Medical Center Rotterdam The Netherlands; ^9^ European Institute for the Biology of Ageing, University Medical Center Groningen University of Groningen Groningen The Netherlands; ^10^ Nuffield Department of Population Health University of Oxford Oxford UK

**Keywords:** diet, genetics, gut microbiome, meta‐analysis, population cohort, TMAO

## Abstract

Trimethylamine *N*‐oxide (TMAO) is a circulating microbiome‐derived metabolite implicated in the development of atherosclerosis and cardiovascular disease (CVD). We investigated whether plasma levels of TMAO, its precursors (betaine, carnitine, deoxycarnitine, choline), and TMAO‐to‐precursor ratios are associated with clinical outcomes, including CVD and mortality. This was followed by an in‐depth analysis of their genetic, gut microbial, and dietary determinants. The analyses were conducted in five Dutch prospective cohort studies including 7834 individuals. To further investigate association results, Mendelian Randomization (MR) was also explored. We found only plasma choline levels (hazard ratio [HR] 1.17, [95% CI 1.07; 1.28]) and not TMAO to be associated with CVD risk. Our association analyses uncovered 10 genome‐wide significant loci, including novel genomic regions for betaine (6p21.1, 6q25.3), choline (2q34, 5q31.1), and deoxycarnitine (10q21.2, 11p14.2) comprising several metabolic gene associations, for example, *CPS1* or *PEMT*. Furthermore, our analyses uncovered 68 gut microbiota associations, mainly related to TMAO‐to‐precursors ratios and the Ruminococcaceae family, and 16 associations of food groups and metabolites including fish‐TMAO, meat‐carnitine, and plant‐based food‐betaine associations. No significant association was identified by the MR approach. Our analyses provide novel insights into the TMAO pathway, its determinants, and pathophysiological impact on the general population.

## Highlights

- Exploration of microbiome‐related metabolites (trimethylamine *N*‐oxide [TMAO], choline, betaine, l‐carnitine, and deoxycarnitine) in 7834 participants from five population cohorts.
- Cardiovascular risk was associated with elevated choline concentrations, but not with TMAO concentrations.
- Characterization of the genetic architecture behind metabolite concentration variability.
- Identification of gut microbial taxonomic abundance associated with metabolite's plasma concentration levels.
- Fish intake is the major dietary driver of TMAO concentrations, and betaine is related to grains and vegetable intake.

## Graphical abstract

Measurements of trimethylamine *N*‐oxide and its precursors in plasma samples from five population cohorts identified an association between choline concentrations and cardiovascular disease. The interindividual variability of metabolite concentrations was determined by common genetic variation, dietary patterns, and the gut microbial composition of the population.

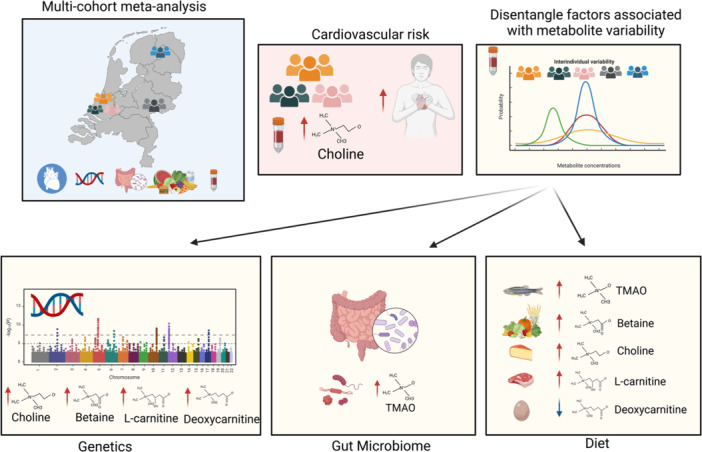

## INTRODUCTION

There is a growing interest in the role of gut microbiome‐related metabolites in cardiovascular disease (CVD) [1, 2]. Trimethylamine *N*‐oxide (TMAO), in particular, has received a lot of attention as a potential promoter of CVD and atherosclerosis [1]. Elevated fasting plasma levels of TMAO have been associated with increased risk for CVD and mortality independently of traditional risk factors in clinical studies [3, 4, 5, 6, 7, 8]. However, investigations on TMAO have mainly been conducted in individuals with a high risk of CVD, existing disease, or multimorbidity while studies on the general population are comparatively scarcer [9, 10, 11, 12]. Proposed mechanisms through which TMAO may promote the development of atherosclerosis and CVD include vascular inflammation, activation of platelets, disturbance of bile acid metabolism, and inhibition of reverse cholesterol transport [13]. Yet, the actual mode of action in disease development may be context‐dependent.

Variation in circulating levels of TMAO is driven by a complex interplay of multiple determinants, such as host genetics, gut microbiome, diet, and kidney function [14]. TMAO can be acquired directly from the diet from fish but it is mainly produced by gut microbiota from the dietary precursors such as choline, l‐carnitine, the carnitine‐derived metabolite, deoxycarnitine (also known as γ‐butyrobetaine), and betaine [15]. Gut microbiota converts these ubiquitous dietary components into trimethylamine (TMA) which is subsequently absorbed from the intestine, transported to the liver, and oxidized into TMAO by hepatic flavin monooxygenases (FMOs), followed by its distribution to different tissues or kidney clearance [16]. However, the extent to which consumption of different animal‐based foods may affect plasma TMAO levels is debated [17, 18, 19], and TMA producers in the human gut microbiome have not been well‐characterized. Association studies point to several genera; however, published results are, in most cases, heterogeneous among studies [15, 20, 21, 22]. At the same time, human genetic variation also contributes to TMAO variability. Rare genetic mutations in the *FMO* type 3 gene (*FMO3*) have been shown to affect the oxidation of TMA to TMAO [23]. However, the role of common genetic variation in TMAO homeostasis remains to be elucidated. As identifying potential drivers for alterations in circulating TMAO levels could have preventive and therapeutic implications for CVD [24], a number of studies have explored determinants of TMAO [15, 21]. However, these cross‐sectional multiomics studies lack sample sizes and are often limited to exploring TMAO variability while overlooking its precursors. The physiological impact of choline and betaine associations with CVD risk remains controversial [25, 26, 27, 28]. Importantly, the major sources of intraindividual variability of these metabolites, together with carnitine and deoxycarnitine, remain unknown.

In addition, CVD risk has often been regarded as a sex‐related disease, with clear prevalence differences between males and females [29]. However, sex differences in the effect of TMAO metabolites on CVD risk remain understudied.

The objectives of this study are twofold: First, we aim to explore the relationships between plasma levels of TMAO and its precursors (betaine, carnitine, deoxycarnitine, choline) and various clinical outcomes, encompassing CVD and mortality. In contrast to much of the existing research conducted within clinical cohorts, our approach involves the utilization of population data derived from 7834 participants enrolled in five prospective Dutch cohorts. By doing so, we aim to shed light on whether elevated concentrations of these metabolites pose a risk to the general population. Second, we attempt to unravel the sources of interindividual variability in these metabolites within the general population. To achieve this, we harness host genetic information, gut microbiome composition, and dietary patterns obtained from distinct subsets of participants within the same five prospective studies.

## RESULTS

### Characteristics of the study population

**Figure 1:**
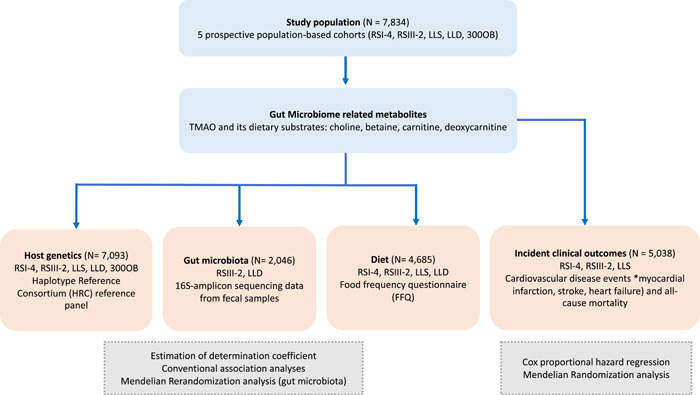
Overview of study design and performed analyses. The study population included participants from Rotterdam Study I‐4 (RSI‐4), Rotterdam Study III‐2 (RSIII‐2), Leiden Longevity Study (LLS), LifeLines‐DEEP (LLD), and 300‐Obese cohort (300‐OB).

An overview of the study design is depicted in Figure 1. Our study population included 7834 participants from five Dutch prospective cohort studies including the Rotterdam Study I‐4, Rotterdam Study III‐2, Leiden Longevity Study (LLS), LifeLines‐DEEP (LLD), and 300‐Obese cohort (300‐OB). A description of the contributing cohorts is provided in Supplementary Material and descriptive characteristics of the study participants are shown in Table 1. The mean age of study participants ranged from 43.4 years (standard deviation [sd] = 14.2) in the LLD to 75.1 years (sd = 6.1) in the Rotterdam Study I‐4. The sex ratio of participants was roughly balanced, with slightly more females in most of the participating studies (up to 58%), with the exception of 300‐OB in which the majority of participants were males (55%). Detailed information on clinical outcomes such as CVD and mortality, host genetics, gut microbiome composition, and diet was available (Figure 1).

**Table 1:** Descriptive characteristics of the study population.

	Rotterdam Study I‐4	Rotterdam Study III‐2	LLS	LLD	300‐OB
*N*	2556	1377	2158	1650	302
Age (years), mean (sd)	75.14 (6.08)	62.68 (5.82)	59.12 (6.71)	43.94 (14.15)	67.05 (5.39)
Women, *N* (%)	1486 (58.14)	748 (54.32)	1208 (55.98)	833 (57.52)	167 (55.29)
Smoking, *N* (%)	301 (12.05)	238 (17.32)	241 (11.17)	289 (19.63)	26 (8.63)
Diabetes, *N* (%)	346 (13.61)	123 (8.94)	90 (4.17)	28 (1.7)	37 (12.25)
Hypertension, *N* (%)	2182 (85.60)	752 (54.77)	159 (7.37)	321 (19.47%)	175 (57.94)
LDLC (mmol/L), mean (sd)	1.63 (0.44)	1.76 (0.51)	1.60 (0.47)	3.14 (0.91)	4.13 (0.96)
HDLC (mmol/L), mean (sd)	1.40 (0.28)	1.37 (0.35)	1.49 (0.33)	1.52 (0.40)	1.33 (0.31)
Serum triglycerides (mmol/L), mean (sd)	1.38 (0.57)	1.33 (0.62)	1.55 (0.82)	1.16 (0.86)	1.83 (1.02)
Lipid‐lowering medication, *N* (%)	583 (22.90)	362 (26.51)	207 (9.59)	29 (2.55)	83 (27.48)
BMI (kg/m^2^), mean (sd)	27.43 (4.13)	27.44 (4.50)	25.43 (3.57)	25.30 (4.22)	30.73 (3.48)
eGFRa^a^	79.41 (13.10)	95.31 (9.29)	94.88 (11.60)	73.13 (12.6)	80.4 (15.68)
Incident cardiovascular disease	544	9	85	23	—
Follow‐up time cardiovascular disease, mean (years)	8.62 (3.49)	2.48	10.7 (1.77)	—	—
Incident mortality events	1295	37	209	—	—
Follow‐up time mortality, mean (years)	10.15 (3.88)	2.48 (1.17)	12.74 (2.22)	—	—

Abbreviations: 300‐OB, 300‐Obese cohort; BMI, body mass index; eGFR, estimated glomerular filtration rate; HDLC, high‐density lipoproteins; LDLC, cholesterol in low‐density lipoproteins; LLD, LifeLines‐DEEP; LLS, Leiden Longevity Study; sd, standard deviation.

^a^ eGFR—calculated using the Chronic Kidney Disease Epidemiology Collaboration equation (CKD‐EPI).

### Incident clinical outcomes are associated with TMAO precursors but not with TMA

Plasma levels of TMAO and its precursors betaine, carnitine, deoxycarnitine, and choline were measured in all participating cohorts (Supporting Information S2: Table S1). We observed weak correlations between TMAO and its precursors (|r| ≤ 0.20) and moderate correlations between precursors (0.30 ≤ |r| ≤ 0.44) (Supporting Information S1: Figure S1).

To assess the relationship between metabolites and incident clinical outcomes including CVD and mortality, Cox proportional hazards regression models with age as a time scale were used. In total, 571 incident CVD events and 1440 mortality events were observed among up to 5011 participants with available covariates from Rotterdam Study I‐4, Rotterdam Study III‐2, and LLS (Supporting Information S2: Tables S2 and S3). A statistically significant association was observed between higher levels of choline and risk of CVD (hazard ratio [HR] 1.17, [95% CI 1.07; 1.28]) after adjusting for sex, body mass index (BMI), hypertension, diabetes, cholesterol in low‐density lipoproteins (LDLC) and high‐density lipoproteins (HDLC), serum triglycerides (TGs), use of lipid‐lowering medication, current smoking, fasting status (if appropriate) (model 1), and multiple testing. The association did not change after further adjustment for the estimated glomerular filtration rate (eGFR) (model 2). The direction of effect size was concordant among the cohorts and no differences were observed between men and women (Supporting Information S2: Table S2). Additionally, a nominal significant association was observed between betaine and risk of CVD (HR 1.10; 95% CI 1.00; 1.21). This association was driven by a strong association in men (HR 1.23; 95% CI 1.07; 1.43). TMAO was associated neither with CVD risk nor mortality. Additional analyses were repeated using concentration quartiles, identifying no significant TMAO association.

To assess the potential causal effect, we performed a Mendelian Randomization (MR) analysis. The results did not provide evidence for any causal effect of TMAO or its precursor metabolites on CVD (Supporting Information S2: Table S4).

### Drivers of variation in gut microbiome‐related metabolite levels

**Figure 2:**
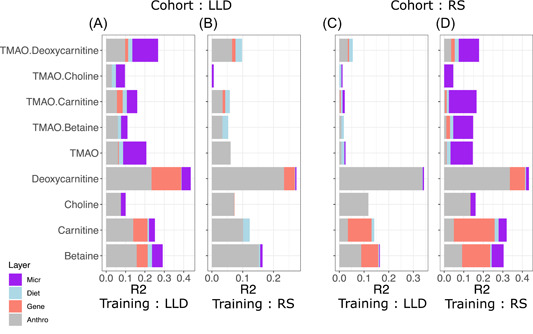
Variance explained in gut microbiome‐related metabolite levels and ratios by different data layers. The *X*‐axis shows the coefficient of determinations *R* ^2^ gained with each additional data layer (gray ‐ anthropometrics; red ‐ genetics; blue ‐ diet; purple ‐ microbiome). Two models were trained, using LLD (A and C) or Rotterdam Study III‐2 (named RS) (B and D) as the train set, and the left‐out cohort as the test set respectively.

We next investigated the sources of variability of each of the measured metabolites. The combined effect of host genetics, gut microbiome, and dietary variation in explaining the variability of plasma metabolite levels and ratios was evaluated by fitting linear regularized additive models (elastic net) on a train set and by estimating the determination coefficient (*R* ^2^) on a test set (see details in 5 section). We trained two models, using LLD or Rotterdam Study III‐2 as the train set and the left‐out cohort as the test set, respectively (Figure 2). Genetic contributions to metabolite variability were large for TMAO precursors but small for TMAO and TMAO‐to‐precursor ratios, although it showed low replicability in the testing cohort. We observed a similar pattern regarding microbial features. For both LLD and Rotterdam Study III‐2‐trained models, a large proportion of TMAO and TMAO ratio variability could be explained by the microbiome in the training set. However, this effect was lost in the test sets. On the other hand, diet showed small but consistent effects between cohorts, while anthropometric effects seemed to be larger in LLD, both in the Rotterdam Study III‐2‐trained and LLD‐trained models.

Subsequently, due to the lack of consistency found in the cohort‐trained models, we sought to identify consistent associations between individual metabolites and host genetics, gut microbiome, or diet through meta‐analysis.

### Genetic variants are underlying levels of TMAO precursors, but not of TMAO itself

**Figure 3:**
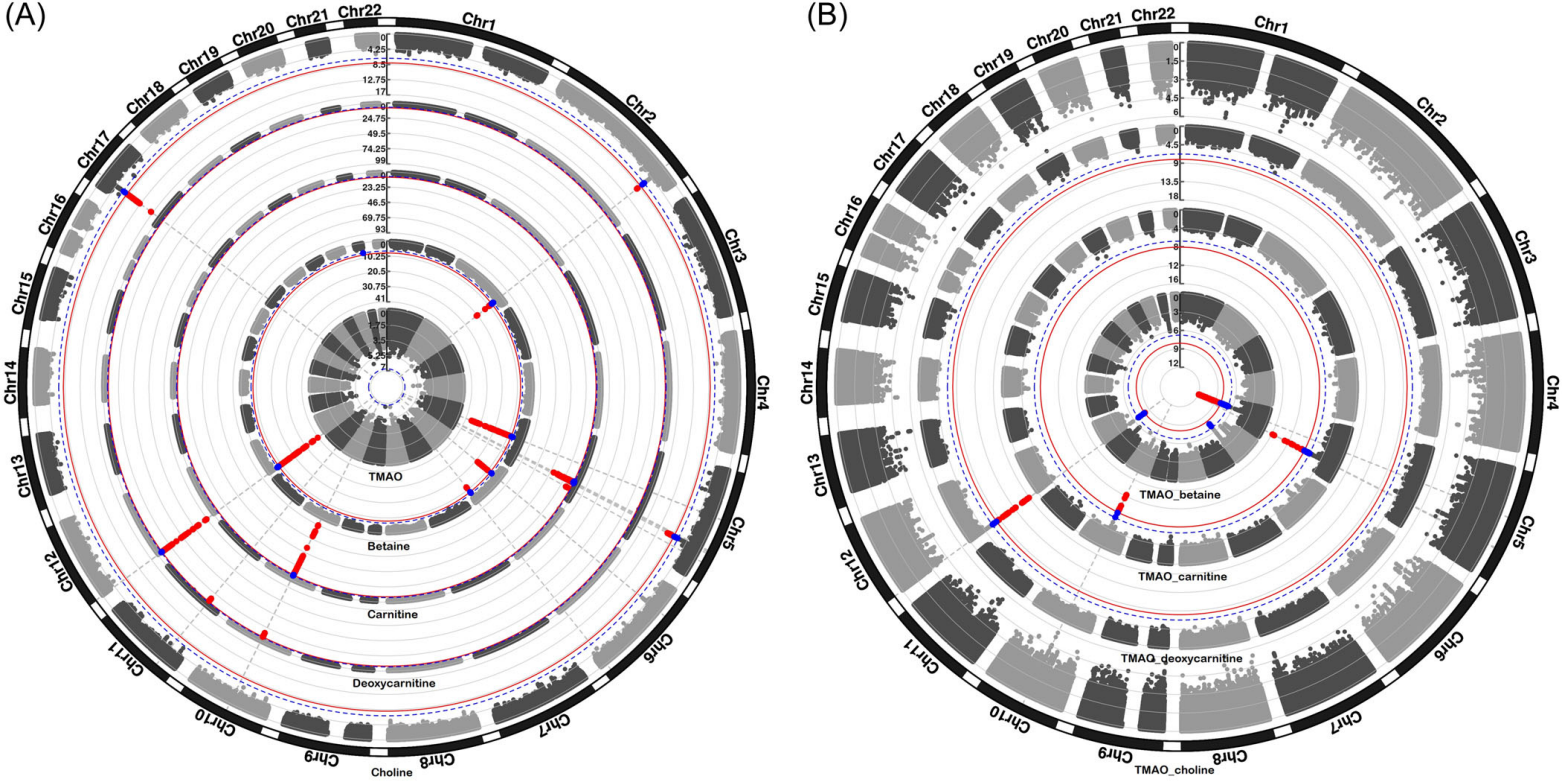
Results of genome‐wide association analyses for (A) individual metabolites and (B) TMAO‐to‐precursor ratios. Each dot represents a genetic variant. Genetic variants surpassing the Bonferroni corrected significance threshold (*p* value < 8.33 × 10^−9^) are highlighted in red. Genetic variants showing suggestive evidence of association (*p* value < 1.7 × 10^−7^) are highlighted in blue.

To evaluate host genetic determinants of TMAO‐related metabolites and ratios, we performed a genome‐wide association study (GWAS) (*N* = 7093) (Figure 3A). The quantile–quantile plots indicated that any cryptic relatedness and/or population stratification were well‐controlled after genomic correction (*λ* ranged between 1.00 and 1.02) (Supporting Information S1: Figure S2, Supporting Information S2: Table S5). Meta‐analyses identified 55 independent genetic variants mapped to five genomic regions for betaine, 89 independent genetic variants mapped to three genomic regions for carnitine, 10 mapped to three genomic regions for choline, and 37 mapped to three genomic regions for deoxycarnitine (Bonferroni corrected genome‐wide significance level, *p* value* *< 8.33 × 10^−9^) (Supporting Information S2: Tables S6–S8). Of these genomic regions, two genomic regions for betaine (6p21.1, 6q25.3), two for choline (2q34, 5q31.1), and two for deoxycarnitine concentration (10q21.2, 11p14.2) have not been reported in previous association studies (Supporting Information S2: Table S7). GWAS of TMAO revealed no genetic variant associated with TMAO concentration at a genome‐wide significant level (Figure 3A), in line with previous studies [30, 31, 32, 33]. As this might suggest that genetic variants have a weak effect on variation in TMAO levels, we combined our results with the results from Hartiala et al. [32] to increase our sample size and improve power. However, no difference in signal was observed. Similarly, an exploration of genetic common and rare variants in the *FMO* gene yielded no significant associations (minimum *p* value > 0.1), in contrast to previous findings [34]. On the other hand, GWAS of TMAO‐to‐precursor ratio revealed genetic loci associated with TMAO to betaine ratio (*n* = 2), TMAO to carnitine ratio (*n* = 2), and TMAO to deoxycarnitine ratio (*n* = 1) (*p* value < 8.33 × 10^−9^) (Figure 3B, Supporting Information S2: Table S9), all of which are overlapping with the precursor's findings.

The genetic variants found have previously been associated with various metabolic (e.g., plasma cholesterol and triglycerides levels, metabolite levels, liver enzyme levels), anthropometric (e.g., height, waist circumference, BMI), and medical traits (e.g., chronic kidney disease, diabetes mellitus, blood pressure) (Supporting Information S2: Table S10).

Furthermore, we performed a sex‐stratified analysis (*N*_females_ = 4026, *N*_males_ = 3067). All significant associations observed in males and almost all significant associations found in females were also significant in the overall analysis (Supporting Information S1: Figure S3, Supporting Information S2: Table S11). An exception was the intronic variant mapped to the *RP1* gene which showed a significant association with TMAO in females (beta = −0.29, *p* value = 2.63 × 10^−9^) but not in males (beta = 0.02, *p* value = 0.78) or the overall analysis. This variant showed heterogeneity between males and females (*p* value = 3.35 × 10^−5^). The *RP1* gene has been reported to function in photoreceptor differentiation (GeneCards Version 3: the human gene integrator).

Gene‐based association analysis revealed 11 genes associated with betaine, 16 with carnitine, six with deoxycarnitine, and two with choline at the gene‐wide significance level (*p* value < 4.42 × 10^−7^) (Supporting Information S2: Table S12). No significant gene sets were identified in the gene‐set analysis (Supporting Information S2: Table S13).

### Heritability estimates and genetic correlation

Single nucleotide polymorphism (SNP)‐based heritability of metabolites was estimated in a range from 0.16 (standard error [SE] = 0.07) for choline to 0.28 (SE = 0.08) for betaine using linkage disequilibrium (LD) score regression (Supporting Information S1: Figure S4a). An overlap of lead genetic loci was observed between betaine and choline (2q34), betaine and deoxycarnitine (12p13.33), carnitine and deoxycarnitine (10q21.2), and carnitine and choline (5q31.1) (Supporting Information S1: Figure S4b). Additionally, we examined genetic overlap on a genome‐wide level by computing genetic correlations. Evidence of suggestive genetic overlap was observed for TMAO and choline (*ρ*_genetic_ = 0.63, *p* value = 2.77 × 10^−2^) and betaine and choline (*ρ*_genetic_ = 0.54, *p* value = 5.7 × 10^−3^), while evidence of significant genome‐wide genetic overlap was observed between TMAO and TMAO‐to‐precursor ratios (Supporting Information S2: Table S14).

### Microbial taxa are associated with plasma levels of TMAO, but not with those of its precursors

**Figure 4:**
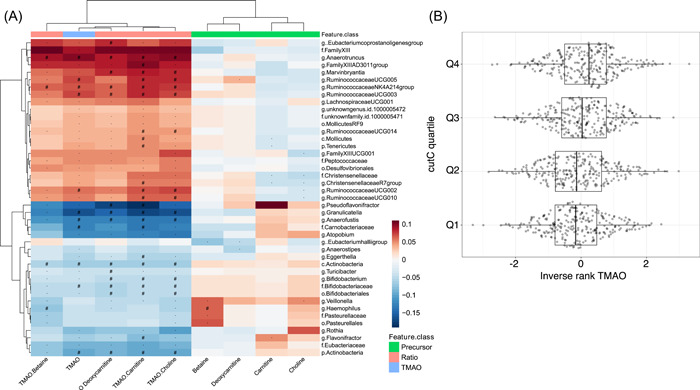
Microbial associations with plasma metabolic concentrations (A) Heatmap showing results of the association analysis between metabolites and gut microbial taxa. Displayed results are after adjustment for age, sex, BMI, and study‐specific covariates. Metabolites are displayed on the *x*‐axis and gut microbial taxa are shown on the *y*‐axis. Red color denotes positive associations and blue color stands for negative associations. Hash symbol (#) represents the Bonferroni significant associations (*p* value < 6.2 × 10^–5^), while star denotes suggestive associations (*p* value < 1.2 × 10^–3^). (B) Boxplots displaying the distribution of normalized TMAO concentration per clr‐transformed abundance quartiles from cutC, measured from metagenomic shotgun sequencing in LLD.

Next, we conducted a meta‐analysis between 241 relative abundances from gut taxa and plasma metabolite concentrations (Figure 4A) identifying 68 associations (Bonferroni multiple test threshold *p* value < 6.21 × 10^−5^). Association effect sizes across cohorts were generally concordant, with no evidence of heterogeneity in most associations (Supporting Information S2: Table S15). Significant associations were predominantly seen for TMAO (11/68) and TMAO‐to‐precursor ratios (56/68) (Supporting Information S2: Table S15). From those, the TMAO/carnitine ratio was associated with an abundance of 23 microbial taxa and the TMAO/choline ratio with 15 microbial taxa. The top associated taxa corresponded to several *Ruminococcaceae* genera (NK4A21, UCG003, UCG005), which were positively associated with TMAO abundance ratio to its precursors, carnitine and choline. Other top associations included the class *Actinobacteria*, which was consistently negatively associated with TMAO‐precursor ratios. The only significant association not directly related to TMAO was a positive association between the genus *Haemophilus* and betaine. Some members of this taxonomic group are able to oxidize choline to generate betaine, although we did not observe a negative association between these taxa and plasma choline [35]. Interestingly, most of the significant or close to significant TMAO associations showed a different direction of effect than associations to at least one precursor (Supporting Information S1: Figure S5). This might highlight taxa with the potential to metabolize TMAO precursors into TMA.

Sex‐stratified analyses identified four significant results in women (Supporting Information S2: Table S16) and four in men (Supporting Information S2: Table S17) (Supporting Information S1: Figure S6). If we focus on suggestive associations in one of the stratified analyses based on sex (*p* value < 1.2 × 10^−3^) but not found to be associated in the overall analysis (*p* value > 1.2 × 10^−3^), we could identify 14 associations, 10 of which showed a significant heterogeneity (*p* value < 0.05) between women and men (Supporting Information S1: Figure S7). The most noteworthy heterogeneous association is between *Coprococcus* and carnitine, for which a clear negative association is seen in males (beta = −0.0178, *p* value = 0.039), which does not appear in females (beta = 0.006, *p* value = 0.583). This taxon has previously been linked to be sex‐linked in mice and pigs [36, 37].

In addition, we leveraged metagenomic shotgun sequencing data available for LLD to quantify specific gene pathways previously related to TMA metabolism from dietary precursors. We quantified a total of 624 proteins belonging to glycine betaine/carnitine transport cluster (gbu A, B, and C), in addition to other known TMA metabolic genes, as choline trimethylamine‐lyase (cutC), choline trimethylamine‐lyase activating enzyme (cutD), the carnitine monooxygenase oxygenase subunit (YeaW), and l‐carnitine/deoxycarnitine antiporter (caiT) gene families. In total, we observed a positive significant association between the abundance of cutC and TMAO (Figure 4B) concentrations and TMAO‐to‐precursor ratios (Supporting Information S2: Table S18). This indicates that this gene might be the major contributor to TMAO variability, or that the major contributors using the other genes were not present in the UniRef90 database we used to extract the gene sequences.

To examine causality, we performed two‐sample MR between taxa and metabolites (excluding ratios). However, we failed to find any significant association (Supporting Information S2: Table S19).

### Diet is associated with plasma levels of TMAO and its precursors

Using 4685 samples with food frequency questionnaires (FFQs) belonging to all but 300‐OB studies, we ran correlations between metabolites and dietary data categorized into 13 major food groups while adjusting for age, sex, and BMI (Supporting Information S1: Figure S8, Supporting Information S2: Table S20). There were 16 correlations that surpassed the Bonferroni corrected threshold for multiple testing (*p* value < 7.58 × 10^−4^). Top positive correlations were observed between TMAO or TMAO‐to‐precursor ratios and fish intake. A significant correlation between fish and vegetable intake led to a correlation between TMAO and vegetable intake, which was not significant if fish intake was accounted for. Betaine levels were positively associated with grains, vegetables, and nuts, while carnitine levels showed a positive association with meat, and negative correlations were observed between cheese intake and choline, and egg intake and deoxycarnitine. Overall, our findings are in line with previous work [14, 26, 38].

## DISCUSSION

We have performed an in‐depth study to identify the potential relationship between plasma levels of TMAO and its precursors (betaine, carnitine, deoxycarnitine, and choline) and various factors including clinical outcomes, host genetics, gut microbiota composition, and diet in up to 7834 participants from five prospective cohort studies mainly based on the general Dutch population.

We observed a significant association between plasma choline concentrations and higher CVD risk. Higher circulating levels of choline correlated with a 17% increase in risk for CVD events. Choline is an essential nutrient that plays a role in various metabolic processes such as C1 metabolism and the synthesis of phospholipids. As such, choline metabolism interacts with the pathways of insulin sensitivity, fat deposition, and energy metabolism [39]. The relationship between dietary choline intake and circulating levels of free choline and its metabolites is concealed by homeostatic regulations and rapid tissue uptake, resulting in a narrow concentration range [39]. Despite being a common dietary compound, we did not identify a strong predictive potential of diet in plasma choline levels. This might be related to the fact that blood samples in our study were mainly collected after overnight fasting. Previous studies reported associations of circulating levels of choline with a higher risk of CVD, mortality and with some traditional cardiovascular risk factors including lower HDL, higher systolic pressure, and triglycerides [4, 8, 20, 40, 41]. However, there are also studies focusing on dietary choline intake with conflicting results. Higher dietary intake of choline was not predictive of incident coronary heart disease or CVD mortality [25, 26, 27, 28]. However, a systematic review and meta‐analysis of six prospective studies reported an association between dietary choline intake and incident CVD [41]. Inconsistency in findings may be related to differences in dietary patterns, sample sizes, follow‐up periods, and geographical location. We also investigated the causality of our association by means of an MR analysis, which failed to support a causal relationship, as seen in previous work [42]. Future studies should focus on improving the strength of the instrumental variables for plasma levels of choline.

In contrast to circulating choline, we did not find an association between plasma TMAO levels and CVD risk or mortality in our population‐based cohorts. Even though a number of studies have shown a relationship between TMAO levels and CVD risk, these results are mostly derived from individuals with a high risk of CVD, existing disease, or multimorbidity [6, 7, 8, 43, 44, 45, 46]. In line with our results, a few population‐based studies did not identify an association between TMAO and cardiometabolic markers, carotid intima media thickness, CVD events including heart attack and stroke and mortality, either [9, 21, 47, 48], while some other studies did report significant associations [10, 11, 12]. To the best of our knowledge, no apparent factor can explain this between‐study heterogeneity, such as sample size, concentration levels, ethnic groups, follow‐up times, and employed methods. These factors were comparable between negative and positive studies. Taken together, we believe that the predictive value of TMAO in CVD onset is still not robust, and further studies need to be done to address its complex interaction with diet and gut microbiome, to understand the existent inter‐study heterogeneity.

Next, we aimed to understand what factors are the most important drivers of metabolite variability in the general population. Exploring the role of host genetics in underlying plasma levels of TMAO and its precursors revealed genome‐wide significant variant associations with plasma levels of TMAO precursors but not for TMAO itself. We confirmed some of the previously reported associations implicated in determining plasma levels of all TMAO precursors. The *DMGDH, BHMT*, and *BHMT2* genes, mapped to the 5q14.1 region, have previously been linked to betaine concentration [49, 50]. These genes are involved in betaine metabolism which is related to a series of interlinking metabolic pathways that include the methionine and folate cycles [51]. Choline is also related to this pathway and we confirmed association with the *PEMT* gene (17p11.2) encoding an enzyme critical in phosphatidylcholine synthesis [52]. Furthermore, the *SLC6A13* gene, mapped to the 12p13.33 locus, has previously been linked to deoxycarnitine levels while the *SLC16A9* gene, mapped to the 10q21.2 locus, has been associated with carnitine levels [53]. Interestingly, we have identified the 10q21.2 region as a novel genetic region underlying deoxycarnitine levels. The lead variant of this region mapped to the *SLC16A9* gene which is involved in urate metabolism. *SLC16A9* encodes a membrane transporter and is expressed in the intestine (GTEx Analysis Release V8), which might indicate a role in deoxycarnitine absorption. Previous studies showed that deoxycarnitine is an intermediary metabolite produced from carnitine by gut microbiota, but it may also act as a precursor to carnitine synthesis [54]. Interestingly, we identified a novel genetic locus in the GWAS of deoxycarnitine that depicts this process. More specifically, the top lead intronic variant of the 11p14.2 locus was mapped to the *BBOX1* gene which is known to catalyze the formation of carnitine from deoxycarnitine and is therefore involved in the carnitine synthesis pathway.

Additionally, we identified the 6p21.1 region as a novel region associated with betaine levels. This region has previously been associated with stroke and type 2 diabetes [52, 53]. Genetic variants in linkage disequilibrium (*r* ^2^ > 0.8) with our lead variant were associated with differential expression of *GNMT* and *PEX6* genes. Interestingly, the *GNMT* gene is involved in the metabolism of methionine. Among novel regions, we have also identified the 2q34 locus underlying the plasma concentrations of choline. The lead genetic variant of this region is mapped to the *CPS1* gene which is involved in the urea cycle. Variants in this gene have previously been linked to creatinine, glycine, betaine and homocysteine levels, BMI, systolic blood pressure, and cholesterol levels [49, 55, 56, 57, 58, 59].

Although we were not able to detect genetic variants underlying plasma TMAO concentrations, we estimated that 20% of genetic variability in TMAO concentration could be explained by common genetic variants. To discover genetic determinants of TMAO, future studies should further increase sample size and focus on complex genetic effects. Additionally, diet intervention studies might be of help, as these could decrease the variability attributed to gut microbiome and diet.

Microbial abundance was mainly associated with TMAO and TMAO‐to‐precursor ratios, which may be interpreted as a proxy for microbial conversion rates. Dietary precursors, on the other hand, did not show strong microbial associations. Several of the taxa we identified have previously been linked to TMAO in European, North American, and Asian studies. For instance, among the taxa belonging to the positively associated cluster, *Ruminococcus* or uncultured *Ruminococcaceae* have frequently been described to correlate with TMA and/or TMAO concentrations in mice and humans [20, 60, 61, 62]. A member of *Family XIII* was associated with TMAO concentrations in mice [63]. *Anaerotruncus* was seen to be decreased upon resveratrol treatment in a mouse model and has been linked to TMAO metabolism [61]. In the same study, several bacterial taxa were increased after resveratrol treatment, including *Bifidobacterium, Bifidobacteriaceae*, and *Bifidobacteriales*, which are negatively associated with TMAO in the present study, also in agreement with other observations in humans [15]. Interestingly, the strongest negative associations were found between TMAO‐to‐precursor ratios and *Pseudoflavonifractor* or *Granulicatella* genera, which have not been reported before. Conversely, other taxa that are often linked to TMAO [64] did not show any significant association in our study, including *Clostridia* or *Escherichia* genera. *Desulfovibrionales*, although did not pass the Bonferroni corrected *p‐*value threshold, showed consistent positive associations with TMAO.

In addition to gut microbiota, diet composition was observed to be an important determinant of metabolite concentrations. For instance, TMAO concentrations and TMAO‐to‐precursor ratios showed a positive association with fish intake. Previous studies linked TMAO concentrations to fish intake and this association has been demonstrated to vary throughout populations and/or regions [15]. Fish consumption was associated with TMAO concentrations in Asian countries and some European countries, while intake of eggs and red meat showed a stronger correlation with TMAO in the population of the United States [17, 18, 19, 65]. In our study population, no association was found between TMAO and eggs or red meat. Fish and seafood are rich in TMAO and TMA and they can be directly absorbed without being transformed by gut microbiota [19]. As previous studies linked TMAO to atherosclerosis and CVD, these findings might be counterintuitive as fish is generally accepted to be cardioprotective [66].

Overall, the large sample size, population‐based design, and comprehensive molecular and epidemiological data of our study helped us to investigate the sources of variation of TMAO and its precursor metabolites in the general population and their health‐related consequences. We were able to study not only TMAO but also the compounds implicated in the TMAO biosynthesis pathway. Furthermore, we were also able to improve statistical power and internally cross‐check the findings by combining data from five population‐based studies. However, our study also has limitations. Microbial species genetic variation is known to modulate bacterial‐related metabolites, thus the taxonomic resolution of 16S might not properly reflect the metabolic potential of the present strains [67]. Metagenomic‐shotgun sequencing experiments will be needed to address that level of variation. The cross‐sectional nature of our metabolomics measurements and gut microbiota assessment in our study only allowed us to investigate the relationship between the two at one time point. To complement our findings and advance our understanding, future studies should focus on assessing longitudinal changes. For instance, a 1‐year follow‐up study in the general population reported large variations in plasma TMAO concentrations, which might underlie the heterogeneous associations related to this metabolite [68].

## CONCLUSION

In conclusion, our data add up to the mounting evidence of research showing that TMAO is not associated with an increased risk of CVD in the general population, despite earlier evidence suggesting this to be the case among patient groups. However, we did show a significant relation between plasma choline levels and higher CVD risk. Our MR revealed no evidence of a causal link between TMAO or its precursors with incident CVD. Furthermore, we also identified several determinants explaining the variability of TMAO and its precursors' blood levels in humans. Gut microbiome was mainly associated with TMAO‐to‐precursor ratios, although the total variability explained of TMAO concentration remains mild and cohort‐specific. Diet was associated with both TMAO and its precursors but could not explain a great proportion of their variation. Genetic contributions to precursor concentrations were greater than to TMAO itself, where no strong genetic effects were seen. The biological mechanisms underlying these associations should be the subject of further studies. Overall, our investigation of the factors determining the interindividual variability of the metabolite concentrations might be used in the future to target interventions aimed at module circulating metabolites. Diet and microbes offer an ethical and effective intervention target that shall be further investigated in follow‐up interventional studies.

## METHODS

### Study population

Our study population included 7834 participants from five cohort studies. Detailed description of participating studies can be found in Supporting Information and descriptive characteristics of study participants are shown in Table 1. Each study was approved by ethical committees (please see Supporting Information for details). Written informed consent was obtained from all participants.

### Metabolite profiling

TMAO and its precursors betaine, carnitine, deoxycarnitine, and choline were quantified in plasma samples of participants from five cohorts by using the liquid chromatography tandem mass spectrometry (LC–MS/MS) method. A detailed description of the method can be found elsewhere [33]. Briefly, before introducing the sample to the mass spectrometer, an analytic column with a C_18_ stationary phase was used to realize an online cleanup of it. The analytes were not retained by this stationary phase but important matrix interferences were retained, such as (phosphor–)lipids [69]. The descriptive statistics of metabolites were coherent across the cohorts (Supporting Information S2: Table S1). In addition to individual metabolites, ratios of TMAO to its dietary precursors were also calculated.

### Incident clinical outcomes

The Rotterdam Study I‐4, Rotterdam Study III‐2, and LLS cohorts had data on incident clinical outcomes including CVD and mortality. Incident CVD events were defined as incident stroke, myocardial infarction, angina pectoris, and heart failure according to the codes of International Classification of Disease, 10th edition. Incident CVD events were assessed continuously through an automated digital linkage of the study database to medical records maintained by general practitioners in the Rotterdam Study and from general practitioner records in the LLS [70, 71]. Information on vital status is additionally obtained from the central registry of the municipality of the city of Rotterdam, and in January 2021, the vital status for participants of the Leiden Longevity Study was updated through the Personal Records Database which is managed by the Dutch governmental service for identity information (https://www.government.nl/topics/personal-data/personal-records-database-brp) [72].

### Baseline clinical characteristics

The baseline clinical characteristics were obtained by means of interviews, physical examination, blood sampling, or medical records from the general practitioner. Assessment of current smoking status, weight, height, blood pressure, glucose concentrations, total LDLC and HDLC, serum TG, creatinine, and medication use including lipid‐lowering medication and use of medication indicated for the treatment of diabetes. Diabetes was defined as fasting glucose concentrations above 7 mmol/L, nonfasting glucose concentrations above 11.1 mmol/L (only if nonfasting concentrations were unavailable), or use of medication indicated for the treatment of diabetes [73], or through medical records from general practitioners. Hypertension was defined as systolic blood pressure ≥140 mmHg, diastolic blood pressure ≥90 mmHg, or the use of medication for the treatment of hypertension [73], or through medical records from the general practitioner. BMI was calculated as weight in kilograms divided by the square of height in meters. Quantified creatinine was used to calculate the eGFR using the Chronic Kidney Disease Epidemiology Collaboration equation (CKD‐EPI) [74].

### Genotyping and imputation

Details on genotyping platforms, calling methods, and quality control (QC) procedures in participating studies are shown in Supporting Information S2: Table S20. Commercially available genotyping arrays were used for genotyping. Similar QC procedures were applied in each study before genotype imputation (Supporting Information S2: Table S20). Genotypes in each cohort were imputed by the Haplotype Reference Consortium reference panel on a Michigan Imputation Server [75].

All genomic coordinates were lifted to Human Build GRCh37/hg19.

### Microbiome processing

The Rotterdam Study III‐2 and LLD cohorts had 16S‐amplicon sequence data available from fecal samples matching plasma collection. Fecal sample collection and 16S sequencing was described elsewhere [76, 77]. In brief, DNA was isolated from the fecal samples belonging to the Rotterdam Study III‐2 cohort, and the V3 and V4 variable regions of the 16S rRNA gene were amplified and sequenced on the Illumina MiSeq sequencer. Similarly, DNA was isolated from fecal samples of LLD participants and the 16S V4 region was sequenced at the Broad Institute using Illumina MiSeq. 16S rRNA data were processed as previously described in a large 16S meta‐analysis including both cohorts [78]. In brief, samples were rarified to 10,000 reads. Reads were classified to a given taxonomic level (genus to phyla) using the RDP classifier (v2.12) [79]. Reads below 0.8 posterior probability to belong to a given taxonomic level were discarded. For each sample, each taxonomic level was centered log‐ratio (clr) transformed. Only taxa observed in above 10% of participants per cohort were used for association resulting in 241 microbial taxa.

### Dietary assessment

The Rotterdam Study cohorts, LLS and LLD had data on dietary intake collected via a validated FFQ. Data on dietary intake in the LLS, LLD, and Rotterdam Study cohorts were collected via validated FFQs [80, 81, 82]. The FFQs assess the frequency of consumption of food items and the number of servings per day. Additionally, information on portion size, type of food item, and preparation methods was collected. The average daily energy and nutrient intake were calculated using the Dutch Food Composition Database. Specific food items were aggregated into food groups in grams per day. The major food groups overlapping between the cohorts were used for subsequent analysis including vegetables, fruit, grains, nuts, eggs, fish, meat, poultry, processed meat, cheese, milk, yogurt, and total dairy products.

#### Incident clinical outcomes analysis

Metabolites were transformed using rank‐based inverse normal transformation. The relationship between metabolites and incident clinical outcomes was assessed using Cox proportional hazards regression models with age as a time scale. The analyses were adjusted for sex, BMI, hypertension, diabetes, LDLC, HDLC, TG, lipid‐lowering medication use, current smoking, and fasting status (if appropriate) (model 1). Subsequently, the associations were adjusted for eGFR (model 2). The proportional hazard assumption was checked using statistical tests incorporated in the *survival* package. Violation of this assumption was observed for some of the covariates and was resolved by stratification. In the LLS, Cox‐type random effect (frailty) regression models were used to adjust for family relations. All analyses were performed using *R*.

The summary statistics results of participating studies were combined using inverse variance‐weighted fixed‐effect meta‐analysis in METAL [83]. The heterogeneity of effects was assessed by *I* ^2^ which indicates the percentage of variance in the meta‐analysis attributable to study heterogeneity [74]. To model the correlation between metabolites, we first used the method of Li and Ji to calculate the number of independent tests [84]. The Bonferroni corrected significance threshold was calculated based on the number of independent tests and set at 0.05/6 independent metabolites = 8.33 × 10^−3^. Additionally, all analyses were stratified by sex. The same steps were followed for the overall analysis.

The potential causal effect of metabolites on clinical CVD outcomes was assessed by MR analysis using the TwoSampleMR package. GWAS summary statistics results were obtained from a large meta‐analysis comprising coronary artery disease cases and controls of UK Biobank resource and CARDIoGRAMplusC4D [85]. Genetic variants with *p* value < 1 × 10^−5^ were used as instruments. Independent genetic variants were selected based on *r* ^2^ in European reference data. The results were kept if these were based on at least three shared genetic variants. Causality was estimated using various MR methods including inverse variance weighted (IVW), MR‐Egger, Wald ratio, Weighted median, Simple Mode, and Weighted Mode.

#### Estimation of determination coefficient in different data layers

The Rotterdam Study cohorts and LLD were used to estimate the total determination coefficient (*R* ^2^) from each of the analyzed data layers in each of the metabolites or metabolite ratios. We used features present in both cohorts including anthropometric covariates (age, sex, BMI), eight overlapping dietary items, 242 bacterial taxonomic abundances, and the number of suggestive genetic variants (*p* value < 1 × 10^−5^) from a meta‐analysis of the GWAS results from LLS and 300‐OB. These two cohorts were used for preselecting variants and were not used to train the model or estimate *R* ^2^. Taxa‐abundance was clr‐transformed, while metabolites and diet were inverse‐rank normal transformed.

We trained a regularized additive linear model, elastic net (glmnet v4.0), and selected the best combinations of hyperparameters alpha (regularization mix) and lambda (regularization strength) through a five‐repeated 10‐fold cross‐validation procedure using the root mean square error as a performance metric (caret v6.0, tunelength = 10). We trained a model for each metabolite (or metabolite ratio) using two different training sets, a training set consisting of the LLD cohort (784 samples) and a training set consisting of the Rotterdam Study III‐2 cohort (772 samples). For the test set (LLD in the Rotterdam Study III‐2‐trained or the Rotterdam Study III‐2 in the LLD‐trained model), the determination coefficient (*R* ^2^) was estimated in nested models. To estimate anthropological *R* ^2^, all other coefficients were made 0. To estimate genetics *R* ^2^, all nongenetics, nonanthropological covariate coefficients were made 0. This was followed by the addition of non‐0 diet parameters and finally the complete model including microbial features. Individual layer *R* ^2^ was quantified by subtracting the *R* ^2^ from the nested models, for example, microbial *R* ^2^ was estimated by subtracting the complete model *R* ^2^ and the diet model.

#### Genome‐wide association analysis

Each participating study performed genome‐wide association analysis under an additive model using metabolites as a dependent variable and variant allele dosage as a predictor. Before the analysis, metabolites were transformed using rank‐based inverse normal transformation. The association analysis was adjusted for age, sex, fasting status if applicable, familial relatedness if appropriate, and principal components if needed. Study‐specific details on covariates and software used to run the analysis are provided in Supporting Information S2: Table S20. The QC was performed using a standardized protocol provided by Winkler et al. [86]. Genetic variants with minor allele count below 10 and low imputation quality (*r* ^2^ < 0.3) were excluded. The summary statistic results were combined using fixed‐effect meta‐analysis in METAL. To account for a small amount of population stratification or unaccounted relatedness, genomic control was applied. After meta‐analysis, genetic variants that were present in less than three participating studies were filtered out. The Bonferroni corrected genome‐wide significance threshold was set at 5 × 10^−8^/6 independent metabolites = 8.33 × 10^−9^. Additionally, the analyses were stratified by sex. The same QC steps were followed for the overall analysis. The sex‐stratified summary statistic results were combined using fixed‐effect meta‐analysis in METAL while applying genomic control. Test statistics of each variant were tested for heterogeneity between males and females.

#### Functional mapping, annotation, and enrichment analysis

Functional Mapping and Annotation of genetic associations (FUMA) was used to characterize genomic loci [87]. Genetic variants that passed the Bonferroni corrected genome‐wide significance threshold and were independent of each other (*r* ^2^ < 0.6) were defined as independent genetic variants. Independent significant genetic variants with *r* ^2^ < 0.1 were defined as lead genetic variants. Independent significant genetic variants with *r* ^2^ ≥ 0.1 or that were 250 bp or closer were assigned to the same genomic risk locus. Each locus was represented by the top lead genetic variant with a minimal *p* value in the locus. Functional annotation was performed using Combined Annotation Dependent Depletion [88], HaploReg [89], and RegulomeDB [90] tools as implemented in FUMA.

Genome‐wide summary statistics were used to perform gene‐based analysis using multi‐marker analysis of genomic annotation as implemented in FUMA. Genetic variants were assigned to the genes from Ensembl build 85 based on genomic location. All genetic variants mapped to the protein‐coding genes were tested for association with metabolites using the SNP‐wide mean model. 1000G phase 3 was used as a reference panel to calculate LD across SNPs and genes. To account for multiple testing, Bonferroni correction was calculated, and the gene‐wide significance threshold was set at 0.05/(18 861 tested genes × 6 independent metabolites) = 4.42 × 10^−7^. Subsequently, gene sets enrichment analysis was also performed using FUMA. Hypergeometric tests were performed to test if genes of interest are overrepresented in any of the 15,496 predefined gene sets obtained from MsigDB. Multiple test correction was calculated based on the total number of gene sets (*p* value = 0.05/15,496 = 3.23 × 10^−6^).

#### Heritability estimates and genetic correlation

The heritability of metabolites and the genetic correlation between them were estimated from GWAS results using the LD Score Regression approach [91]. We used precomputed LD scores for Europeans. Only genetic variants available in HapMap3 were used.

#### Gut microbial taxa and metabolites association analysis

Each of the metabolites and TMAO‐to‐precursor ratios were rank‐based inverse normal transformed. Standard linear regression models were carried out to associate the transformed metabolite and taxonomy abundances while adjusting for age, sex, BMI, and study‐specific covariates (sample batch, time in mail, and storage time in the Rotterdam Study III‐2). This analysis was also reproduced in sex‐stratified samples. Common taxonomy associations in the Rotterdam Study III‐2 and LLD were meta‐analyzed using a fixed‐effects, inverse‐variance analysis (R package meta v4.12). The association's heterogeneity was measured by Cochran's *Q* statistic.

To correct for multiple testing, we determined the number of independent tests using the method of Li and Ji [84]. There were 134 independent tests among microbial taxa and six independent tests among metabolomic measures. The Bonferroni significance threshold was set at 0.05/(134 independent microbial taxa × 6 independent metabolites) = 6.2 × 10^−5^, while a suggestive threshold was set at 1/(134 × 6) = 1.2 × 10^−3^.

The potential causal relation between gut microbial taxa and metabolite levels was tested by two‐sample MR using the TwoSampleMR package. Only taxa‐to‐metabolite associations that surpassed our significant thresholds (*p* value < 6.2 × 10^−5^) were considered for the analysis. We used the GWAS summary statistics for metabolites produced in this work, while for microbial taxa we obtained summary statistics from a large 16S meta‐analysis comprising 18,340 individuals from 24 cohorts including both Rotterdam Study III‐2 and LLD[88]. Genetic variants with *p* value < 1 × 10^−5^ were selected as instruments. Bacterial taxa with no available instruments were removed from the analysis. Independent genetic variants were selected as instrumental variables based on an *R* ^2^ threshold of 0.001 (1000 Genomes in the European reference population). The number of instruments varied between 11 and 20. The causality was estimated using various MR methods including IVW, MR‐Egger, Wald ratio, Weighted median, Simple Mode, and Weighted Mode. In addition, we also assessed genetic variant heterogeneity and evidence of horizontal pleiotropy (using Egger). Individual summary statistics for genetic variants were estimated using Wald ratio tests.

To investigate the correlation of distinct microbial pathways implicated in the metabolism of TMA from dietary precursors [92, 93], including the glycine betaine/carnitine transport cluster (gbuA, gbuB, and gbuC), cutC, cutD, the YeaW, and caiT, we conducted an analysis based on 845 matching annotations extracted from UniRef90 (June 2019). For the analysis, we utilized the remaining UniRef90 data set as a background universe. A database of unique oligopeptides was constructed using ShortBRED (v 0.9.4) [94] to enable unambiguous mapping of reads for subsequent quantification.

Metagenomic shotgun sequencing data from the LifeLines‐DEEP cohort [95] was obtained for further analysis (EGA accesion: EGAC00001000457). Preprocessing of shotgun data was carried out using publicly available pipelines (https://github.com/SegataLab/preprocessing). Specifically, we employed Trim Galore (v0.6.6) to remove low‐quality reads (<Q20), reads with lengths <75 bp, and those with more than two ambiguous nucleotides. Host DNA contamination was mitigated by aligning reads to the hg19 human genome using Bowtie2 (v2.3.4.3) [96]. Subsequently, forward and reverse reads were merged, and gene quantification was performed using ShortBRED. This approach yielded the quantification of 624 proteins (caiT = 96, cutC = 81, cutD = 109, GbuA = 86, GbuB = 39, GbuC = 184, YeaW = 29). Normalization of read numbers per marker length was carried out, and the total abundance per gene family was computed. To address data compositionality, we applied the clr‐transformation. Correlation analysis between gene family clr‐transformed abundances and (inverse‐ranked) metabolite concentrations was performed using a unique multivariable model while accounting for age and sex. To control for multiple testing, we implemented the Benjamini‐Hochberg False Discovery Rate procedure.

#### Diet and metabolites correlation analysis

Each food group, metabolite and TMAO‐to‐precursor ratio were rank‐based inverse normal transformed before analysis. Partial correlation coefficients were calculated between each transformed food group item and metabolite or metabolite ratio while adjusting for age, sex, and BMI. Summary statistics results of participating studies were combined by performing a fixed‐effect meta‐analysis in METAL. To model the correlation between food groups, the method of Li and Ji was used to calculate the number of independent tests. Associations were considered significant if they surpassed the Bonferroni corrected significance threshold of 0.05/(11 independent food groups × 6 independent metabolite) = 7.58 × 10^−4^.

## AUTHOR CONTRIBUTIONS

Conception and design of the study: Sergio Andreu‐Sánchez, P. Eline Slagboom, Cornelia M. van Duijn, Jingyuan Fu, Dina Vojinovic. Collection of the data: Alexander Kurilshikov, Marian Beekman, Mohsen Ghanbari, Martijn van Faassen, Inge C. L. van den Munckhof, Marinka Steur, Amy Harms, Thomas Hankemeier, M. Arfan Ikram, Maryam Kavousi, Trudy Voortman, Robert Kraaij, Mihai G. Netea, Joost H. W. Rutten, Niels P. Riksen, Alexandra Zhernakova, Folkert Kuipers, P. Eline Slagboom, Cornelia M. van Duijn, Jingyuan Fu. Analysis: Sergio Andreu‐Sánchez, Shahzad Ahmad, Dina Vojinovic. Interpretation of the data: Sergio Andreu‐Sánchez, Shahzad Ahmad, Alexander Kurilshikov, Marian Beekman, Trudy Voortman, P. Eline Slagboom, Cornelia M. van Duijn, Jingyuan Fu, Dina Vojinovic. Drafting of the manuscript: Sergio Andreu‐Sánchez, Dina Vojinovic. All authors have read the final manuscript and approved it for publication.

## CONFLICT OF INTEREST STATEMENT

The authors declare no conflict of interest.

## ETHICS STATEMENT

**Leiden Longevity Study (LLS)**

In accordance with the Declaration of Helsinki, we obtained informed consent from all participants before they entered the study. Good clinical practice guidelines were maintained. The study protocol was approved by the ethical committee of the Leiden University Medical Center before the start of the study (P01.113).

**LifeLines‐DEEP (LLD)**

The Lifelines study was approved by the ethics committee of the University Medical Center Groningen (METc2007/152). All participants signed an informed consent form before enrollment. The LLD study was approved by the Institutional Ethics Review Board of the University Medical Center Groningen (ref. M12.113965), the Netherlands.

**Rotterdam Study (RS)**

The Rotterdam Study has been approved by the Medical Ethics Committee of the Erasmus MC (registration number MEC 02.1015) and by the Dutch Ministry of Health, Welfare and Sport (Population Screening Act WBO, license number 1071272‐159521‐PG). The Rotterdam Study Personal Registration Data collection is filed with the Erasmus MC Data Protection Officer under registration number EMC1712001. The Rotterdam Study has been entered into the Netherlands National Trial Register (NTR; www.trialregister.nl) and into the WHO International Clinical Trials Registry Platform (ICTRP; www.who.int/ictrp/network/primary/en/) under shared catalog number NTR6831. All participants provided written informed consent to participate in the study and to have their information obtained from treating physicians.

**300 Obesity (300‐OB) cohort**

The 300OB study was approved by the IRB CMO Regio Arnhem‐Nijmegen (number 46846.091.13).

## Supporting information


**Figure S1**: Phenotypic correlation between metabolites included in the analyses.
**Figure S2**: Quantile–quantile plots for genome‐wide association meta‐analyses.
**Figure S3**: Results of sex‐stratified genome‐wide association analysis for individual metabolites.
**Figure S4**: SNP‐based heritability and overlap of lead genomic loci identified in the GWAS of TMAO and its precursors.
**Figure S5**: Top associations of microbial taxonomy with metabolites.
**Figure S6**: Results of association analysis between metabolites and gut microbial taxa in males and females.
**Figure S7**: Gender‐heterogeneous associations of microbial taxonomy with metabolites.
**Figure S8**: Results of correlation analysis between metabolites and ratios and food groups.

**Table S1**: Descriptive characteristics of metabolites and quantification method.
**Table S2**: Results of association analysis between metabolites and incident cardiovascular disease events. The analyses were performed for overall sample and females and males separately. The associations that surpassed significance threshold (*p* value < 8.33 × 10^−3^) are highlighted in bold.
**Table S3**: Results of association analysis between metabolites and mortality. The analyses were performed for overall sample and females and males separately. The associations that surpassed significance threshold (*p* value < 8.33 × 10^−3^) are highlighted in bold.
**Table S4**: Mendelian randomization analysis evaluating TMAO and its precursors with respect to cardiovascular disease. b, effect; se, standard error; pval, *p* value; nsnps, number of SNPs acting as instrumental variables.
**Table S5**: Genomic inflation factor for the individual studies.
**Table S6**: Genome‐wide significant results (*p* value < 8.33 × 10^−9^) for genome‐wide association study of TMAO, its precursors betaine, carnitine, choline and deoxycarnitine and TMAO‐to‐precursor ratios. Associations with HetPval > 0.1 are listed in the table.
**Table S7**: Index of lead genetic variants and genomic loci these variants are assigned to (novel findings highlighted).
**Table S8**: All genetic variants in linkage disequilibrium with any of independent significant genetic variants and their functional annotation.
**Table S9**: Independent lead genetic variants and loci these variants are mapped to in TMAO‐to‐precursor ratio GWAS.
**Table S10**: Pleotropic associations for independent significant genetic variants and all genetic variants in LD with these.
**Table S11**: Genome‐wide significant findings in sex‐stratified analyses.
**Table S12**: Results of gene‐based analysis. Listed associations passed gene‐wide significance level (*p* value < 4.42 × 10^−7^).
**Table S13**: Results of gene‐set analysis that showed suggestive evidence of association with metabolites (*p* value = 1/(15485*6) = 1.08 × 10^−5^).
**Table S14**: Results of genome‐wide genetic overlap between metabolites and metabolite ratios. Associations with evidence of significant genome‐wide genetic overlap were shown in bold.
**Table S15**: The results of association analyses between metabolites and gut microbial taxa.
**Table S16**: The results of association analysis between metabolites and gut microbial taxa in females.
**Table S17**: The results of association analysis between metabolites and gut microbial taxa in males.
**Table S18**: Association between TMAO metabolic pathway abundances, using shotgun sequencing data from LLD and metabolite concentrations.
**Table S19**: Mendelian randomization analysis evaluating TMAO and its precursors with regard to gut microbiota. b, effect; se, standard error; pval, *p* value; nsnps, number of SNPs acting as instrumental variables.
**Table S20**: Results of correlation analyses between TMAO, its precursors, TMAO‐to‐precursor ratios and food groups.
**Table S21**: Information on genotyping and analysis.

## Data Availability

The data are available from the author on request. All relevant data supporting the key findings of this study are available within the article and its supplementary information files. Other data are available from the corresponding author upon reasonable request. Due to ethical and legal restrictions, individual‐level data from the Rotterdam Study (RS) cannot be made publicly available. Data are available upon request to the data manager of the Rotterdam Study Frank van Rooij (f.vanrooij@erasmusmc.nl) and subject to local rules and regulations. This includes submitting a proposal to the management team of RS, where upon approval, analysis needs to be done on a local server with protected access, complying with GDPR regulations. The use of Lifelines data and materials must comply with the informed consent signed by Lifelines participants specifying that their collected data will not be used for commercial purposes. The raw 16S amplicon sequencing, metabolomics, and basic phenotype data (age, sex, and BMI) are deposited in the EGA database with the study ID EGAS00001001704 (https://ega-archive.org/studies/EGAS00001001704), which includes Data set ID EGAD00001003453 for raw sequencing data and data set for metabolomics data. There is a minimal access procedure for the access of the EGA data set that includes a contact address and an online data access form https://goo.gl/forms/TWHlrmbXaXNqWnnl2, which is very simple and is only intended to ensure that the data is being requested for research/scientific purposes only. Submitted data access forms will be evaluated by the data manager and Lifelines. For requests from verified academic parties, access will be granted within 2 weeks. For requests from commercial parties, Lifelines will perform a pre‐Data Privacy Impact Assessment (pre‐DPIA) to assess the risks of the proposed processing of personal data (e.g., purpose, storage, access, archiving, etc.) with respect to the GDPR subject rights. Based on the outcome of the pre‐DPIA, Lifelines will decide whether sharing data with the commercial entity is allowed and/or whether additional measures have to be taken. Lifelines diet and genotype information should be directly requested from Lifelines. (1) Data are requested by filling out the application form to request “Available Lifelines‐data” at https://www.lifelines.nl/researcher/how-to-apply/apply-here; (2) Lifelines will evaluate project proposals to ensure compliance with the Lifelines data access policy, informed consent of Lifelines participants and the GDPR, and that the data is being requested for noncommercial research; (3) Upon approval, Lifelines will send Data and Material Transfer Agreement (DMTA) contracts to the applicants; and (4) After the required contracts are signed, Lifelines will provide access to data via the Workspace or HPC and link the raw and processed DMP sequencing data to the Lifelines phenotypes. Lifelines strives to accomplish steps 2–4 at 2 weeks per step, assuming that no extra actions by the applicant or Lifelines are required. The source data of the Leiden Longevity Study are not automatically publicly available due to legal and informed consent restrictions. Reasonable requests to access the data sets should be directed to the PI of the Leiden Longevity Study P. Eline Slagboom (p.slagboom@lumc.nl) and Marian Beekman (m.beekman@lumc.nl). If data access is approved, the data users are bound by a data access agreement. This includes responsibilities with respect to third party data sharing and maintaining participant privacy. Further responsibilities include a responsibility to acknowledge data sharing. The source genetic data of the 300OB cannot be shared. Interested researchers can send a proposal to Niels.Riksen@Radboudumc.nl with regard to the genetics data of the 300OB. The scripts used for analysis are saved in GitHub https://github.com/GRONINGEN-MICROBIOME-CENTRE/Groningen-Microbiome/tree/master/Projects/TMAO_metagenomics/16S_association. Supplementary materials (figures, tables, graphical abstract, slides, videos, Chinese translated version and update materials) may be found in the online DOI or iMeta Science http://www.imeta.science/.
